# How People Take Cold

**Published:** 1879-03

**Authors:** 


### HOW PEOPLE TAKE COLD.

Not by tumbling into the river and draggling home wet as a drowned rat; not by being pitched into the mud, or spilled out in the snow in sleighing time; not by walking for hours over shoe-top in mud; not by soaking in the rain without an umbrella; not by scrubbing the floor until the un-nameable sticks to you like a wet rag; not by hoeing potatoes until you are in a lather of sweat; not by trying to head a pig in mid-winter, and induce him to run the other way, for he won't do any such thing; not by steaming over the wash-tub; not by essaying to teach Biddy to make mince pies for Christmas, when you don't know how yourself, and then worrying yourself into a perspiration because the pies stuck to the pan, and came out in a muss, forgetting that pie-pans, like people, are rather the better for a little greasing, alias soft soap; these are not the things which give people colds; and yet people are all the time telling us how they "caught their death by exposure." Horace Greeley once said, "O for a leisure week to read books"

